# The co-inoculation of *Pseudomonas chlororaphis* H1 and *Bacillus altitudinis* Y1 promoted soybean [*Glycine max* (L.) *Merrill*] growth and increased the relative abundance of beneficial microorganisms in rhizosphere and root

**DOI:** 10.3389/fmicb.2022.1079348

**Published:** 2023-01-09

**Authors:** Wentao Zhang, Guohao Mao, Jiayao Zhuang, Hao Yang

**Affiliations:** ^1^Collaborative Innovation Center of Sustainable Forestry in Southern China of Jiangsu Province, Nanjing Forestry University, Nanjing, China; ^2^College of Forestry, Nanjing Forestry University, Nanjing, China

**Keywords:** soybean, PGPR inoculant, beneficial flora, rhizosphere, root, soil improvement

## Abstract

Currently, plant growth-promoting rhizobacteria (PGPR) microbial inoculants are heavily used in agricultural production among which *Pseudomonas* sp. and *Bacillus* sp. are two excellent inoculum strains, which are widely used in plant growth promotion and disease control. However, few studies have been conducted on the combined use of the two bacteria. The aim of this study was to investigate the effects of co-inoculation of these two bacteria on soybean [*Glycine max* (L.) *Merrill*] growth and physiological indexes and further study the effect of microbial inoculants on native soil bacterial communities and plant endophyte microbiota, especially microorganisms in rhizosphere and root. A pot experiment was conducted and four treatments were designed: group without any strain inoculant (CK); group inoculated with *Pseudomonas chlororaphis* H1 inoculant (J); group inoculated with *Bacillus altitudinis* Y1 inoculant (Y) and group inoculated with equal volume of *P. chlororaphis* H1 inoculant and *B. altitudinis* Y1 inoculant (H). Compared with CK, the three inoculant groups J, Y, and H exhibited improved soybean growth and physiological indexes, and group H was the most significant (*p* < 0.05). In terms of rhizosphere bacterial community structure, the relative abundance of native *Luteimonas* (9.31%) was higher in the H group than in the J (6.07%), Y (3.40%), and CK (5.69%) groups, which has potential value of disease suppression. Besides, compared with bacterial communities of the other three groups in soybean roots, group H increased the abundance of beneficial bacterial community for the contents of *Allorhizobium-Neorhizobium-Pararhizobium-Rhizobium*, *Devosia*, and *Methylobacillus* significantly increased (*p* < 0.05). In conclusion, we found that the composite inoculum of *Pseudomonas chlororaphis* H1 and *Bacillus altitudinis* Y1 could effectively promote soybean growth, increase yield and improve the beneficial bacterial community in root and rhizosphere and have certain value for soil improvement.

## 1. Introduction

As the main source of edible oil in China, the market demand for soybean [*Glycine max* (L.) *Merrill*] remains tremendous. According to statistics, the national soybean production in 2021 was only 16.4 million tons, accounting for only 10% of the total supply (Ren et al., 2021). Improving the autonomous yield and quality of soybean has become a major challenge for sustainable agricultural development in China. The development and application of new microbial inoculants, represented by PGPR inoculants, may be an effective solution (Andy et al., 2020).

Plant growth-promoting rhizobacteria (PGPR) refer to a diverse community of free-living soil bacteria that colonize and promote plant growth in the rhizosphere, thereby increasing crop yield (Mhatre et al., 2019). Studies have reported that PGPR can affect plant growth in several ways, including the production of phytohormones such as IAA (Sachdev et al., 2009; Perez-Flores et al., 2017), iron carriers (Rahimi et al., 2020), and ACC deaminase (Shahzad et al., 2013; Brunetti et al., 2021); promotion of uptake of soluble mineral elements such as K and P from the environment (Grobelak et al., 2018; Bisht et al., 2019); formation of symbiotic nitrogen (N) fixation systems with the plant body (Senthilraja et al., 2013; Gómez-Godínez et al., 2019); and production of pathogen antagonists to control pests and diseases (Tabatabaei and Saeedizadeh, 2017; Zhao et al., 2022). PGPR is widely distributed in soil and has a large host range. Many species of PGPR have been successfully inoculated into common crops, including wheat (Islam et al., 2014), rice (Pramanik et al., 2018), oilseed rape (Tulumello et al., 2021), maize (Costa-Gutierrez et al., 2020), and soybean (Masciarelli et al., 2014), making PGPR a powerful tool in agricultural biotechnology.

Through the identification of microbial diversity in PGPR, studies have reported that *Bacillus* sp. and *Pseudomonas* sp. are the most common genera that can be isolated from plant rhizosphere (Chaturvedi and Singh, 2016). Among them, *Bacillus* sp. is the most dominant bacterial genus in soil, which produces thermotolerant and-resistant bacilli such as *B. cereus*, in addition to its diverse growth-promoting mechanisms (Huang et al., 2021). It is widely used in the development of microbial fertilizers, for example, *B. thuringiensis* is used for the eradication of pine nematodes (Guo et al., 2022) and *B. subtilis* is used for inhibiting cotton yellow leaf disease (Zhao et al., 2022). In addition, some species of *Pseudomonas* sp. have been reported to have roles in N fixation and plant growth (Liu et al., 2017), pest control (Ma et al., 2018; Príncipe et al., 2018), heavy metal enrichment (Qian et al., 2022), and pesticide degradation (Wang et al., 2017). In the future, PGPRs such as *Bacillus* and *Pseudomonas* have great potential as a novel microbial fertilizer in agricultural production.

Most of the early inoculants were made of single strains, which had a single function of growth promotion, while the crops have diverse needs for growth promotion. Therefore, the research of microbial inoculants gradually tends to select multi-strain and multi-functional composite inoculant (Baez-Rogelio et al., 2017). For example, the combined use of *Pseudomonas fluorescens*, which can produce plant growth regulators, and *Azospirillum brasilense*, which is a nitrogen-fixing bacterium, significantly promoted the harvest index and rice yield under field conditions, increasing them by 16 and 20.2%, respectively (Inés et al., 2021). It is precisely because co-inoculation of PGPR strains is more reliable and effective than single inoculation in agriculture, which is why we chose the mixed group as one treatment group.

In this experiment, two PGPR strains isolated from the rhizosphere of wild soybean (*Glycine soja Sieb.* and *Zucc.*) were used as the source of the inoculated strains, and the effects of single and combined inoculant treatments on the growth condition, and microbial diversity indices of soybean in rhizosphere were investigated. We hope to increase the abundance of soil beneficial bacterium, such as rhizobia, and reduce the production of soil and plant pathogenic bacteria when bacterial inoculants are used to promote soybean growth and increase soybean production, thus providing a new method for the sustainable development of soybean agriculture.

## 2. Materials and methods

### 2.1. Sample collection and isolation of bacteria

The wild soybean root nodules were collected in an agriculture field in You County, Zhuzhou City, Hunan Province. The plants were uprooted and loosely adhered soil was detached by gentle shaking and collected root nodules were gently rinsed with running water to remove the peripheral soil. Large sized and healthy nodules were selected for bacteria isolation and brought back to the laboratory immediately.

In laboratory, the wild soybean root nodules were soaked in 95% alcohol for 5–10 s, followed by 2.5% sodium hypochlorite for 2 min, and then rinsed with sterilized water (six times), Each surface sterilized nodule was crushed using a sterile glass rod in a sterile test tube containing 1 mL sterile physiological saline (0.8% NaCl). One loopful of the nodule suspension was streaked on plates that contained YEM (yeast extract mannitol) agar medium and incubated in the dark for 3–4 days at 30°C (Wei et al., 2009). Repeat scribing and purification on fresh YEM plates until single colonies are obtained. Bacteria of varying morphology were individually numbered and stored at 4°C in refrigerator for further characterization.

Physicochemical properties of the strains were determined, gram staining, gelatine liquefaction, starch hydrolysis, glucose, lactose, nitrate reduction, Voges Proskauer, methyl red, hydrogen sulfide and citrate tests were detected by the standard methods in Bergey’s Manual of Determinative Bacteriology (Holt, 1994).

### 2.2. Determination of plant growth promoting characteristics

The capacity of the strains to solubilize phosphate was determined in liquid Pikovskaya medium (Pikovskaya, 1948). The ability of antagonistic bacteria to produce siderophore was evaluated on CAS medium (Schwyn and Neilands, 1987). The production of indole-3-acetic acid (IAA) was performed by using Salkowski’s colorimetric method (Loper and Schroth, 1986).

### 2.3. Pot experiment

Before planting, the two strains were separately inoculated into 250 mL Erlenmeyer flasks containing 100 mL of LB liquid medium (He et al., 2020) and incubated at 25°C with shaking (200 rpm) for 48 h. The bacterial cultures were centrifuged at 8,000 *g* for 10 min at 4°C and the collected bacterial cells were resuspended in saline solution (0.85% NaCl) to generate densities of 3 × 10^7^ colony-forming units (CFU/mL) for inoculation, and bacterial inoculants were produced. Subsequently, four different treatments were designed as follows: (1) CK, untreated plants inoculated with 50 mL of saline solution (control), (2) J, plants were inoculated with 50 mL of *Pseudomonas chlororaphis* H1 inoculant, (3) Y, plants were inoculated with 50 mL of *Bacillus altitudinis* Y1 inoculant and (4) H, plants were inoculated with 25 mL of *B. altitudinis* Y1 inoculant and 25 mL of *P. chlororaphis* H1 inoculant. Each treatment was independently replicated three times.

The soil for planting was collected from the experimental field in Nanjing Forestry University with a pH of 7.58 and the contents of available nitrogen (AN), phosphorus (AP), potassium (AK) and organic carbon (SOC) were 186.48 mg·kg^−1^, 10.25 mg·kg^−1^, 79.7 mg·kg^−1^ and 14.10 g·kg^−1^, respectively.^.^ And soil bulk density is 1.15 g·cm^−3^. The soil was sieved (2 mm) and partial disinfection was performed at 121°C for 15 min at 15 psi.

Full and uniform sized seeds of soybean [*Glycine max* (L.) *Merrill*] were selected, sterilized with 1% NaCIO for 5 min, and rinsed three times with sterilized water. Soybean seeds were placed on two sheets of damp filter paper and germinated at 28°C in darkness for 48 h. Afterward, seedlings were transplanted to a plastic pot (30 × 30 × 20 cm; 3 kg sterilized soil per pot; soil and vermiculite in a 3:1 volume ratio) and cultured in a growth chamber at 30°C/20°C (day/night), 75% relative humidity, and 20 h photoperiods (500 μmol/m^2^/s).

Each treatment was independently replicated three times for a total of 12 plastic pots. At the two-true-leaf stage of soybean, bacterial inoculants were inoculated into the soil. Inoculum was applied in the form of root irrigation and distilled water was added daily. The experimental field was located at the College of Forestry, Nanjing Forestry University and the experiment was performed from April 2021 to July 2021. Plant growth indexes were measured after 90 days of plant growth. Each treatment was independently replicated three times.

### 2.4. Determination of soybean growth index, chlorophyll content, and photosynthetic intensity

#### 2.4.1. Biomass and root system assay

The height and stem thickness of the seedlings were measured using a tape and vernier calipers. The root morphology was assessed using a root system scanner (Epsonperfection V700). The aboveground and underground parts of the plants and the root system were weighed after they were dried at 65°C for 24 h. Each treatment was independently replicated three times.

#### 2.4.2. Determination of chlorophyll content of leaves

With the exact area of the template or punch cut 1 cm^2^ leaves (pay attention to avoid the thicker veins), cut into about 5 mm, about 1 mm wide filament. The leaf filaments were put into a graduated test tube containing 5 m L 80% acetone. After sealing the tube, the filaments were extracted in the dark until they turned completely white (overnight). Gently shake the test tube several times in the middle to shorten the extraction time. The optical density (OD) was measured at 663 nm and 645 nm using a spectrophotometer (UV-120, Japan) and the contents of chlorophyll A and B were calculated according to Arnon, (1949) method. Each treatment was independently replicated three times.

#### 2.4.3. Determination of photosynthetic parameters of leaves

Photosynthetic data of soybean leaves were measured using LI-6400 portable Photosynthetic apparatus (LI-Cor Inc., United States). The measured data included Net photosynthetic rate (Pn), stomatal conductance (Gs), intercellular carbon dioxide concentration (Ci) and transpiration rate (Tr). The experiment was conducted from 8 a.m. to 12 a.m. on a sunny day in July. Each treatment was independently replicated three times.

### 2.5. Rhizosphere sampling and measurement of soil physicochemical properties

Three months later (July 2021), rhizosphere samples consisting of loosely adhered to roots were collected, following the final harvest of soybean plants. The collected soil samples were sealed in plastic bags and transferred immediately to the laboratory. To remove plant roots, debris and stones, all samples were sieved (2 mm sieve) and thoroughly homogenized. Subsequently, the soil was divided into two parts: one part was stored in a 4°C refrigerator for soil physicochemical property analysis, another part was sent to Guangzhou Kidio Co. Ltd., for sequencing to analyze soil microbial diversity with the use of Illumina MiSeq platforms at Guangzhou Kidio Co. Ltd. Alpha diversity is the analysis of species diversity in a single sample, including Chao, Shannon, Simpson and other indices. Venn diagrams were used to characterize the bacterial communities shared in all samples (Yang et al., 2017).

Finally, 12 soil samples (4 treatments × 3 replicates) were prepared for soil physicochemical property analysis and another 12 soil samples (4 treatments × 3 replicates) for microbial diversity analysis. Soil available phosphorous (AP) was determined by extraction with 0.5 mol L^−1^ NaHCO_3_ using molybdenum antimony resistance colorimetry (Olsen et al., 1954). Soil hydrolyzed nitrogen (AN) was determined by the alkali-hydrolyzed diffusion method (LY/T 1228–2015; Liu et al., 2020). Available potassium (AK) was extracted using sodium bicarbonate (Olsen’s method; Sun et al., 2018). Organic C was measured using the Walkley–Black dichromate oxidation method (Nelson and Sommers, 1965). Soil pH was measured by using the METTLER TOLEDO pH meter. Bulk density of soil samples at 20 cm depth was measured by using a steel ring of diameter 5 cm, dried at 105°C for 8 h, and weighed (Di et al., 2017).

Each treatment was independently replicated three times.

### 2.6. Root sampling and microbial composition analysis

Root sampling and soil sampling were conducted simultaneously to wash away soil particles from the roots. Finally, a total of 12 root samples (4 treatments × 3 replicates) were collected while 0.2 g of roots were selected for endophytic bacterial community analysis. These roots were surface-sterilized by successive immersion in 75% ethanol for 3 min, 1.2% sodium hypochlorite for 3 min, and then 75% ethanol for 1 min, followed by five rinses with sterilized deionized water (Xu et al., 2022). The sterility of the root surface was verified by 16S rRNA gene PCR amplification and plate incubation method (Seghers et al., 2004) using the final rinse water. Surface sterilized root samples were immediately stored at-80°C and later sent to Guangzhou Kidio Co. Ltd. for sequencing to analyze soil microbial diversity with the use of Illumina MiSeq platforms at Guangzhou Kidio Co. Ltd.

### 2.7. Statistical analysis

All graphics were drawn using Origin 9.0 software. Mean and standard deviation for each set of data were calculated. Data related to soil properties, microbial diversity, and abundance were analyzed using Tukey’s HSD test in SPSS 26.0 software with significance level set at *p* < 0.05.

## 3. Results

### 3.1. Identification of isolated strains

A total of 4 strains (H1, Y1, B1, and B2) were isolated through screening (Table 1). The 16S rRNA gene of bacterial isolates revealed amplified product of 1.5 kbp. Further, nucleotide sequencing and BLAST analysis showed similarity of Y1 to *Bacillus altitudinis* 1910ICU267 and *Bacillus altitudinis* SK2-22, H1 to *Pseudomonas* sp. SB955, *Pseudomonas* sp. B17 and *Pseudomonas chlororaphis* BF 2–5, B1 to *Sphingomonas* sp. DX-T3-03 and *Sphingomonas aquatilis* NJ-SWW-8-C, B2 to *Brevundimonas vesicularis* MW2 and *Brevundimonas nasdae* ABL17CA28. For further tests, H1 was named *Pseudomonas chlororaphis* H1 and Y1 was named *Bacillus altitudinis* Y1 and their 16 s rRNA sequences were stored in the NCBI database with the accession numbers ON329818 (H1) and ON247222 (Y1).

**Table 1:** Identification of bacterial isolates based on 16S rRNA gene in NCBI.

Isolate	Accession	Most closely related species	Identity (%)	Taxonomic designation
Y1	ON329818	*Bacillus altitudinis* 1910ICU267	100	*Bacillus* sp.
		*Bacillus altitudinis* SK2-22	100	
H1	ON247222	*Pseudomonas* sp. SB955	100	*Pseudomonas* sp.
		*Pseudomonas* sp. B17	99.9	
		*Pseudomonas chlororaphis* BF2-5	99.9	
B1	OP861542	*Sphingomonas* sp. DX-T3-03	100	*Sphingomonas* sp.
		*Sphingomonas aquatilis* NJ-SWW-8-C	100	
B2	OP861543	*Brevundimonas vesicularis* MW2	100	*Brevundimonas* sp.
		*Brevundimonas nasdae ABL17CA28*	100	

### 3.2. Physicochemical properties and characteristics of strain H1 and Y1

H1 was rod-shaped and gram-negative bacteria and Y1 was rod-shaped and gram-positive bacteria. The biochemical reactions of Voges Proskauer, starch hydrolysis, glucose, citrate, and gelatin liquefaction were all positive and hydrogen sulfide, lactose and methyl red were negative of these two strains (Table 2). Also, nitrate reduction test of H1 was positive while Y1 was negative. In terms of plant growth-promoting ability, *Pseudomonas chlororaphis* H1 produced 33.47 mg/L of IAA and 77.85 mg/L of soluble phosphate was released. *Bacillus altitudinis* Y1 produced 38.49 mg/L of IAA and 152.36 mg/L of soluble phosphate was released. Both of these two strains could produce siderophore.

**Table 2:** Physiological and biochemical properties of *Pseudomonas chlororaphis* H1 and *Bacillus altitudinis* Y1.

Tests employed	H1	Y1
Voges-Proskauer test	+	+
Methyl red test	−	−
Hydrogen sulfide	−	−
Gelatin liquefaction	+	+
Starch hydrolysis	+	+
Nitrate reduction	+	−
Glucose degradation	+	+
Lactose degradation	−	−
Citrate degradation	+	+
Gram reaction	−	+
IAA (mg/L)	33.47 ± 4.11	38.49 ± 2.13
Phosphate solubilization (mg/L)	77.85 ± 6.44	152.36 ± 10.62
Siderophore production	+	+

“+“and “−” indicate positive and negative reactions, respectively.

### 3.3. Effects of inoculants on the growth and yield of soybean

#### 3.3.1. Aboveground and underground biomass of soybean

The four treatments were conducted on soybean, including CK (control group), J (H1 inoculant group), Y (Y1 inoculant group) and H (co-inoculation group). All growth indicators of aboveground biomass of soybean were significantly improved after the J, Y and H treatments compared with the CK treatment. Among them, plant height increased by 11.75, 6.77 and 23.78%; stem thickness increased by 19.72, 5.45 and 33.55% in the J, Y, and H groups, respectively (Table 3). Also, dry weight and fresh weight of aboveground and underground biomass in bacterial inoculant groups increased compared with the CK group, with the most significant increase in the H group (*p* < 0.05). Overall, the promotion of the growth of aboveground and underground biomass of soybean was more pronounced and outstanding in the H group (mixed inoculation) than in the J and Y groups (single inoculation). The order of growth promoting effect was H > J > Y > CK.

**Table 3:** Comparison of growth of soybean plants after treatment with various bacterial inoculants.

	Plant height (cm)	Stem diameter (cm)	Above-ground fresh weight (g)	Above-ground dry weight (g)	Under-ground part fresh weight (g)	Under-ground part dry weight (g)
CK	52.85 ± 1.60b	9.18 ± 0.78c	217.06 ± 11.77b	75.97 ± 6.50b	21.37 ± 2.73c	6.69 ± 0.99b
J	59.06 ± 2.85b	10.99 ± 0.47ab	243.39 ± 20.78ab	85.19 ± 3.24ab	27.61 ± 3.05ab	9.00 ± 1.50ab
Y	56.43 ± 3.07b	9.68 ± 0.10bc	223.43 ± 22.56b	78.20 ± 6.06b	23.47 ± 3.52bc	7.64 ± 0.70b
H	65.42 ± 4.23a	12.26 ± 1.10a	266.71 ± 15.26a	93.35 ± 7.14a	29.59 ± 1.89a	10.24 ± 1.31a

CK represents potted soybean inoculated with 50 mL saline solution. J, Y, and H represent potted soybean inoculated with 50 mL Pseudomonas sp. H1, Bacillus sp. Y1, and complex inoculant, respectively. Data are means ± standard errors (*n* = 3). Different letters represent significant difference at *p* < 0.05 (Tukey’s HSD test).

#### 3.3.2. Root indexes of soybean

Root indexes are shown in Table 4. It can be found that group H was superior to the other three groups in root surface area, root length, total root volume and other root indexes, indicating that mixed inoculation promoted soybean root growth.

**Table 4:** Comparison of root indexes of soybean after treatment with various bacterial inoculants.

	Root length (cm)	Root surface area (cm^2^)	Total root volume (cm^3^)	Tip number	Branch number	Nodule number
CK	413.46 ± 6.41b	121.51 ± 8.95b	2.90 ± 0.44c	2,884 ± 338b	4,474 ± 419b	20.33 ± 5.86b
J	440.03 ± 8.63ab	141.15 ± 12.16ab	4.34 ± 0.29ab	3,209 ± 198ab	5,354 ± 405ab	34.67 ± 5.03a
Y	432.69 ± 11.78bc	126.77 ± 10.72b	3.64 ± 0.55bc	3,066 ± 213b	4,786 ± 523b	28.33 ± 5.51ab
H	457.04 ± 15.91a	158.09 ± 16.25a	4.73 ± 0.25a	3,602 ± 230a	5,772 ± 383a	36.67 ± 4.16a

Data are means ± standard errors (*n* = 3). Different letters represent significant difference at *p* < 0.05 (Tukey’s HSD test).

#### 3.3.3. Photosynthetic strength in soybean

The photosynthetic parameters of soybean leaves under different bacterial inoculum applications were shown in Table 5. Compared with CK, J and Y, the net photosynthetic rate in H was the highest, which increased by 15.44, 5.89 and 14.40%, respectively and reached 34.17 μmol CO_2_/(m^2^·s). Stomatal conductance in H increased compared with the group CK, J, and Y and reached 387.67 mmol CO_2_/(m^2^·s). Compared with the control group, J and Y, the intercellular CO_2_ concentration significantly decreased after adding co-inoculation in H group, which reached 123.67 μmol/mol. The transpiration rate of H was the highest, reaching 6.37 mmol H_2_O/(m^2^·s). In general, H group had the most significant effect on photosynthetic coefficient of soybean leaves.

**Table 5:** Comparison of photosynthetic parameters after treatment with various bacterial inoculants.

	Net photosynthetic rate (Pn)/(μmol·m^−2^·s^−1^)	Transpiration rate (Tr)/(mmol·m^−2^·s^−1^)	Stomatal conductance (Gs)/(mmol·m^−2^·s^−1^)	Intercellular CO_2_ concentration (Ci)/(μmol·mol^−1^)
CK	29.60 ± 1.82b	5.54 ± 0.09b	179.67 ± 17.10c	177.00 ± 20.00a
J	32.27 ± 1.29ab	5.95 ± 0.17ab	275.33 ± 15.65b	134.33 ± 8.35b
Y	29.87 ± 0.31b	5.61 ± 0.18b	163.00 ± 19.47c	187.00 ± 11.15a
H	34.17 ± 1.53a	6.37 ± 0.06a	387.67 ± 26.74a	123.67 ± 7.26b

Data are means ± standard errors (*n* = 3). Different letters represent significant difference at *p* < 0.05 (Tukey’s HSD test).

#### 3.3.4. Chlorophyll content in soybean

As can be seen from Table 6, the photosynthetic pigment content of plants in the three groups with bacterial treatment were higher than those in CK, and the contents of chlorophyll A, B and total chlorophyll in group H were the highest, reaching 2.58, 0.89, and 3.47 mg/g.

**Table 6:** Comparison of Chlorophyll Content of soybean leaf after treatment with various bacterial inoculants.

	Chlorophyll A (mg·g^−1^)	Chlorophyll B (mg·g-1)	Total chlorophyll (mg·g-1)
CK	1.92 ± 0.16c	0.72 ± 0.02b	2.64 ± 0.18c
J	2.26 ± 0.19b	0.75 ± 0.05b	3.01 ± 0.23b
Y	2.06 ± 0.09bc	0.74 ± 0.03b	2.81 ± 0.11bc
H	2.58 ± 0.11a	0.89 ± 0.04a	`3.47 ± 0.12a

Data are means ± standard errors (*n* = 3). Different letters represent significant difference at *p* < 0.05 (Tukey’s HSD test).

#### 3.3.5. Soybean yield

The soybean yield results of the four treatments are shown in Table 7, and group H has the highest yield among the four groups. Compared with CK, J and Y groups, the number of effective pods in H group increased by 44.72, 1.85 and 22.89%. And seed number per plant in H was 135.67, which was 39.87, 4.36 and 18.67% higher than CK, J and Y groups, respectively, and the weight of 100 seeds was 35.59 g, which was 29.14, 9.81 and 9.78% higher than that of CK, J and Y groups, respectively. Finally, yield per plant was 48.54 g, increased by 82.59, 15.65, and 30.10% in the H group compared to the CK, J and Y group, respectively.

**Table 7:** Comparison of soybean yield after treatment with various bacterial inoculants.

	Number of pods	Seed number	100-seed weight (g)	Dry biomass (g)
CK	50.67 ± 9.07b	97.00 ± 14.80b	27.56 ± 1.46b	26.59 ± 2.55b
J	72.00 ± 5.29a	130.00 ± 8.89a	32.41 ± 3.31ab	41.97 ± 2.68a
Y	59.67 ± 8.39ab	114.33 ± 12.42ab	32.42 ± 4.79ab	37.31 ± 8.48ab
H	73.33 ± 1.53a	135.67 ± 9.02a	35.59 ± 4.22a	48.54 ± 8.99a

Data are means ± standard errors (*n* = 3). Different letters represent significant difference at *p* < 0.05 (Tukey’s HSD test).

### 3.4. Effects of plant growth-promoting rhizobacteria inoculants on potted soil physicochemical properties

Four groups of treatments were set up, including control group CK, single inoculant group Y, single inoculant group J, and complex inoculant group H. Compared with the control group CK, soil physicochemical properties were improved in all groups with inoculants (Table 8). Among them, soil available phosphorous (AP) and soil hydrolyzed nitrogen (AN) in group H treated with the complex bacterial inoculant were 13.76 mg·kg^−1^, 490.67 mg·kg^−1^, 156.14 mg·kg^−1^, 19.01 g·kg^−1^, respectively, which significantly increased (*p* < 0.05) in AP, AN, AK and SOC contents compared with control group CK and J and Y single inoculant group, and the pH of soil acidification decreased to a certain extent. Also, bulk density in H group is 0.98 g·cm^−3^, which is the lowest in four groups.

**Table 8:** Comparison of physicochemical properties of soybean potted soil after treatment with different inoculants.

	AP (mg·kg^−1^)	AN (mg·kg^−1^)	AK (mg·kg^−1^)	SOC (g·kg^−1^)	pH	Bulk Density (g·cm^−3^)
CK	10.70 ± 0.71b	374.07 ± 22.77c	115.32 ± 10.03c	16.35 ± 0.58c	7.13 ± 0.11a	1.15 ± 0.01a
J	13.19 ± 0.44a	464.96 ± 30.20a	140.64 ± 6.03ab	17.92 ± 0.37b	6.78 ± 0.13b	1.09 ± 0.06ab
Y	12.61 ± 0.75a	438.19 ± 17.59b	129.68 ± 7.55bc	16.70 ± 0.61c	6.86 ± 0.17ab	1.03 ± 0.01bc
H	13.76 ± 0.47a	490.67 ± 28.29a	156.14 ± 9.34a	19.01 ± 0.25a	6.73 ± 0.20b	0.98 ± 0.01c

Data are means ± standard errors (*n* = 3). Different letters represent significant difference at *p* < 0.05 (Tukey’s HSD test).

### 3.5. Effect of inoculants on bacterial communities in rhizosphere

#### 3.5.1. Alpha diversity

**Figure 1:**
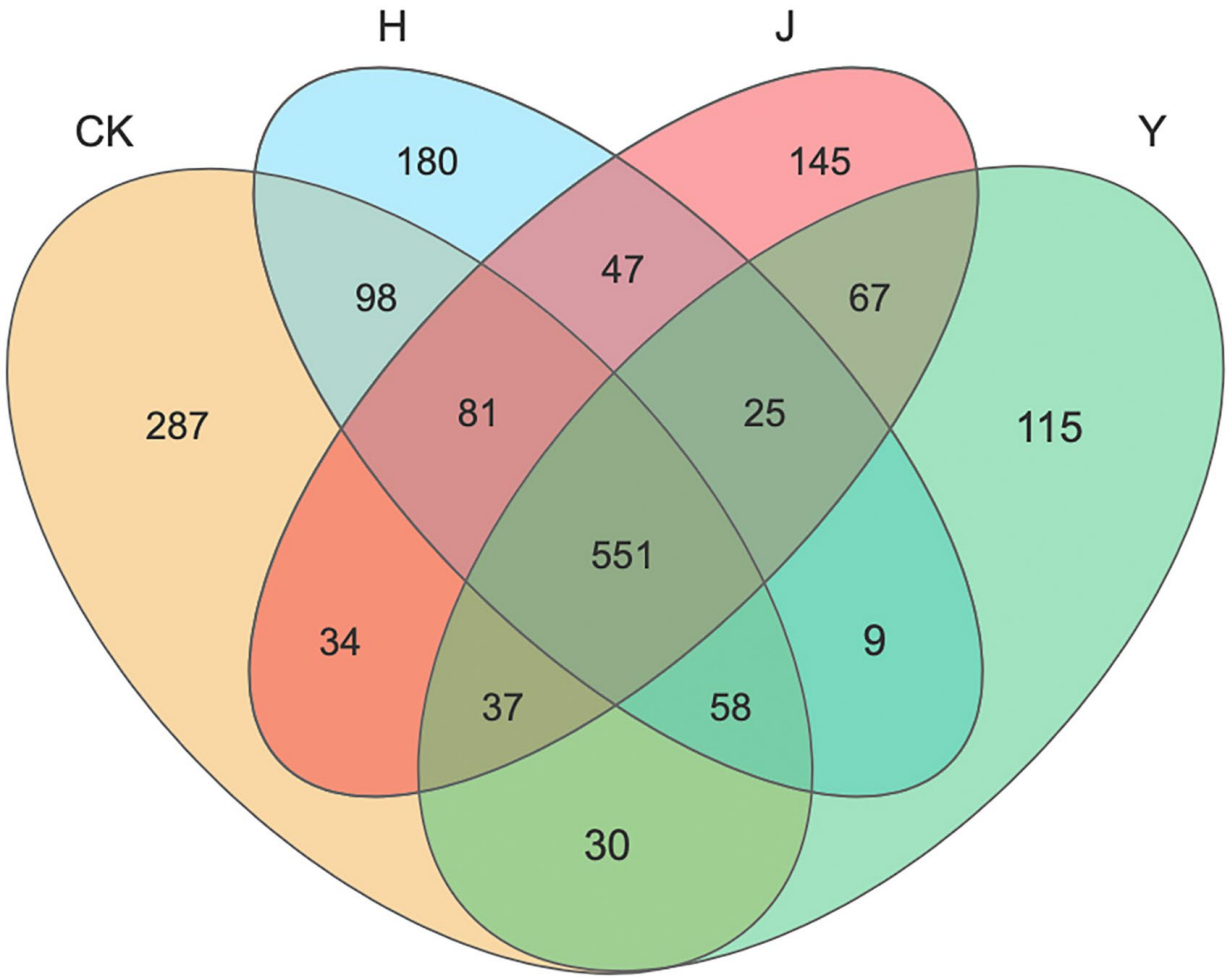
Venn diagram of bacteria in the rhizosphere of various treatment groups. Venn diagram demonstrated the numbers of shared and unique observed OTUs at 97% similarity among various treatments. CK represents soybean-potted rhizosphere soil inoculated with 50 mL saline solution. J, Y, and H represent soybean-potted rhizosphere soil inoculated with 50 mL *Pseudomonas chlororaphis* H1 inoculant, 50 mL *Bacillus altitudinis* Y1 inoculant, and 25 mL *Pseudomonas chlororaphis* H1 inoculant and 25 mL *Bacillus altitudinis* Y1 inoculant, respectively.

Venn diagrams (Figure 1) indicated the number of shared or unique OTUs among soil samples or subpopulations. The number of OTUs in rhizosphere was different under various treatments of bacterial inoculum. For the bacterial community of rhizosphere in potted soybean, there were 551 identical OTUs under the four treatments CK, H, J, and Y, accounting for 46.85, 52.53, 55.82, and 61.77% of the total OTUs in each treatment group, respectively. In addition, the number of OTUs unique to the CK, H, J, and Y treatments was 287, 180, 145, and 115, accounting for 24.40, 17.16, 14.69, and 12.89% of the total OTUs in each treatment group, respectively. The number of OTUs in the samples obtained with various inoculants was in the order CK > H > J > Y. This indicated that the combined inoculants exhibited a greater effect on the bacteria in soybean-potted soil compared with the single inoculants, and the bacterial composition of the soil in the combined inoculant group was more similar to that of the sterile treatment group, whereas the degree of microbial diversity produced by the plants under the combined treatment was higher.

The Sobs, Chao, and ACE indexes mainly reflected the community richness of the samples. These three indexes of the CK group were significantly higher than those of the inoculant groups, indicating that the bacterial community richness of the basin soil was higher in the CK group (Table 9). Simpson’ s and Shannon’s indexes usually reflect the homogeneity and diversity of soil bacterial communities, respectively. Both these indexes exhibited a significant decrease in the inoculant groups, indicating that the homogeneity and diversity of bacterial communities in the CK group were higher than those in the inoculant groups. However, the diversity of the soil samples with the H treatment was significantly higher than that of the soil samples with the J and Y treatments. The diversity indices analysis revealed that both single and mixed inoculants mostly changed the original bacterial levels in the soil, whereas the reduction in bacterial diversity was more pronounced in the single inoculant treatment.

**Table 9:** Bacterial community diversity indices in soybean-potted rhizosphere.

	Sobs	Shannon	Simpson	Chao	ACE	Pielou
CK	1187.00 ± 81.69a	7.53 ± 0.06a	0.99 ± 0.01a	1267.07 ± 80.37a	1318.28 ± 90.03a	0.73 ± 0.02a
J	977.33 ± 59.53b	6.26 ± 0.20b	0.95 ± 0.01b	1060.55 ± 61.66b	1092.03 ± 61.22b	0.63 ± 0.01b
Y	887.53 ± 35.92b	5.36 ± 0.36c	0.88 ± 0.04c	992.35 ± 34.72b	1029.36 ± 32.53b	0.55 ± 0.04c
H	1025.37 ± 86.56b	7.06 ± 0.27a	0.97 ± 0.01ab	1106.78 ± 71.95b	1145.86 ± 71.34b	0.70 ± 0.03a

Data are means ± standard errors (*n* = 3). Different letters represent significant difference at *p* < 0.05 (Tukey’s HSD test).

#### 3.5.2. Bacterial community structure of rhizosphere

**Figure 2:**
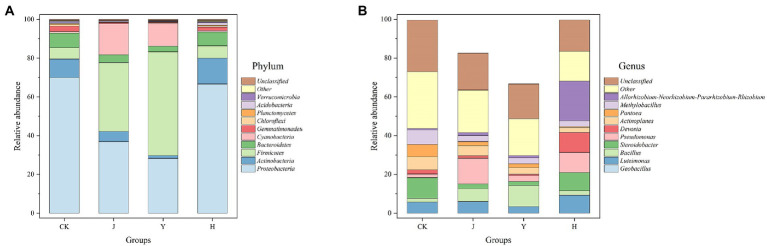
**(A,B)** Comparison of microbial populations at phylum and genus level in rhizosphere. CK represents soybean-potted rhizosphere soil inoculated with 50 mL saline solution. J, Y, and H represent soybean-potted rhizosphere soil inoculated with 50 mL *Pseudomonas chlororaphis* H1 inoculant, 50 mL *Bacillus altitudinis* Y1 inoculant, and 25 mL *Pseudomonas chlororaphis* H1 inoculant and 25 mL *Bacillus altitudinis* Y1 inoculant, respectively.

As shown in Figures 2A,B, the bacterial community structure of the composite (H) group was similar to the CK group in terms of bacterial phylum structure but different in abundance, and differed significantly from the single (J and Y) inoculant treatments.

The top 5 dominant bacterial phyla with maximum abundance after each treatment were *Proteobacteria*, *Actinobacteria*, *Firmicutes*, *Bacteroidetes*, and *Cyanobacteria* (Figure 2A). Compared with the CK group, the relative abundance of *Firmicutes* and *Cyanobacteria* in the J and Y groups increased significantly, whereas that of *Proteobacteria* exhibited a decreasing trend. In addition, the relative abundance of the *Actinobacteria* in group H was higher than other three groups (Table 10).

**Table 10:** Comparison of major bacterial community composition of soybean-potted rhizosphere soil at the phylum level.

	CK	J	Y	H
*Proteobacteria*	69.95 ± 5.30a	37.00 ± 2.41b	28.29 ± 1.81c	66.70 ± 3.27a
*Actinobacteria*	9.63 ± 0.96b	5.16 ± 2.09c	1.43 ± 1.10d	13.31 ± 1.47a
*Firmicutes*	5.91 ± 1.27c	35.54 ± 2.19b	53.57 ± 7.31a	6.36 ± 2.55c
*Bacteroidetes*	7.51 ± 1.50a	4.08 ± 0.83b	2.90 ± 1.49b	7.17 ± 0.94a
*Cyanobacteria*	0.77 ± 0.17b	16.02 ± 3.19a	11.95 ± 4.62a	0.79 ± 0.11b

Bacterial genera with average relative abundance > 0.5% are shown and do not contain unclassified taxa. Data are means ± standard errors (*n* = 3). Different letters represent significant difference at *p* < 0.05 (Tukey’s HSD test).

At the level of known bacterial genera, the top 4 genera in order of maximum abundance were *Geobacillus*, *Steroidobacter*, *Bacillus*, and *Luteimonas* (Figure 2B) in all the treatment groups. The relative abundance of *Geobacillus* was 17.41 and 33.29% in the J and Y groups, respectively, which was significantly higher than that in the CK (0.40%) and H (0.35%) groups. The relative abundance of *Bacillus* was 6.70 and 10.95% in the J and Y groups, respectively, which was significantly higher than that in the CK (1.97%) and H (2.28%) groups (Table 11).

**Table 11:** Comparison of major bacterial community composition of soybean-potted rhizosphere soil at the genus level.

	CK	J	Y	H
*Geobacillus*	0.40 ± 0.08c	17.41 ± 1.11b	33.29 ± 7.25a	0.35 ± 0.10c
*Luteimonas*	5.69 ± 1.20b	6.07 ± 0.25b	3.40 ± 1.10c	9.31 ± 0.66a
*Bacillus*	1.97 ± 0.71c	5.70 ± 0.52b	10.95 ± 0.89a	2.28 ± 1.40c
*Steroidobacter*	4.71 ± 2.07b	2.29 ± 0.85b	2.01 ± 1.14b	9.37 ± 1.10a
*Pseudomonas*	1.85 ± 0.33c	13.71 ± 1.59a	3.09 ± 0.41c	6.68 ± 1.50b

Data are means ± standard errors (*n* = 3). Different letters represent significant difference at *p* < 0.05 (Tukey’s HSD test).

The relative abundance of *Steroidobacter* (9.37%) in the H group was significantly higher than that in the CK (4.71%), J (2.29%), and Y (2.01%) groups. In addition, the relative abundance of *Luteimonas* in H (9.31%) group was higher than that in the CK (5.69%), J (6.07%), and Y (3.40%) groups.

### 3.6. Effects of inoculants on bacterial communities in root

#### 3.6.1. Root alpha diversity

As shown in Table 12, these three indexes of the Sobs, Chao, and ACE indexes of the CK group were significantly higher than those of the three inoculant groups. However, in terms of Simpson, Shannon and Pielou index, CK group was lower than H group and higher than J and Y group, indicating that H group significantly increased (*p* < 0.05) root species diversity index compared with single inoculum group and control group. The order of α-diversity structure in root is H > CK > J > Y.

**Table 12:** Bacterial community diversity indices in soybean-potted root.

	Sobs	Shannon	Simpson	Chao	ACE	Pielou
CK	1348.33 ± 72.01ab	7.17 ± 0.12a	0.98 ± 0.01bc	1527.00 ± 29.43ab	1549.35 ± 38.14ab	0.60 ± 0.01c
J	1286.67 ± 30.92bc	6.94 ± 0.36ab	0.98 ± 0.01ab	1421.77 ± 70.26bc	1459.53 ± 56.84bc	0.67 ± 0.03ab
Y	1250.67 ± 18.58c	6.47 ± 0.17b	0.97 ± 0.01c	1408.15 ± 51.07c	1453.90 ± 38.29c	0.63 ± 0.01bc
H	1357.33 ± 33.50a	7.21 ± 0.24a	1.00 ± 0.01a	1551.34 ± 48.01a	1585.99 ± 42.15a	0.70 ± 0.02a

Data are means ± standard errors (*n* = 3). Different letters represent significant difference at *p* < 0.05 (Tukey’s HSD test).

#### 3.6.2. Bacterial community structure of root

**Figure 3:**
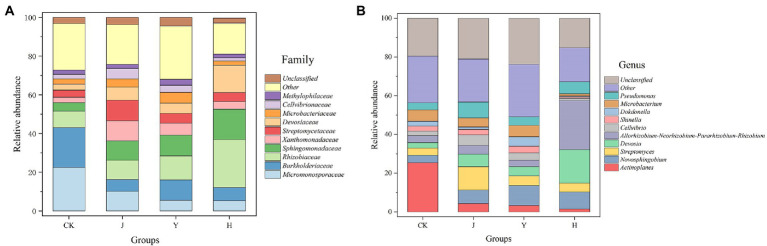
**(A,B)** Comparison of microbial populations at family and genus level in root. CK represents soybean-potted rhizosphere soil inoculated with 50 mL saline solution. J, Y, and H represent soybean-potted rhizosphere soil inoculated with 50 mL *Pseudomonas chlororaphis* H1 inoculant, 50 mL *Bacillus altitudinis* Y1 inoculant, and 25 mL *Pseudomonas chlororaphis* H1 inoculant and 25 mL *Bacillus altitudinis* Y1 inoculant, respectively.

At the family level (Figure 3A), compared with CK in control group, the relative abundance of *Xanthomonadaceae* (3.92%), *Sphingomonadaceae* (15.61%), *Devosiaceae* (14.11%), and *Rhizobiaceae* (24.81%) in group H increased, while *Burkholderaceae* (6.82%) decreased (Table 13). Compared with J and Y, the beneficial bacteria *Sphingomonadaceae Devosiaceae* and *Rhizobiaceae* significantly increased (*p* < 0.05). At the genus level (Figure 3B), compared with CK, J and Y (Table 14), H significantly decreased the content of *Actinoplanes* (1.54%) and the contents of *Allorhizobium-Neorhizobium-Pararhizobium-Rhizobium* (25.41%) and *Devosia* (17.19%) significantly increased (*p* < 0.05), indicating that the co-inoculation of *Pseudomonas chlororaphis* H1 and *Bacillus altitudinis* Y1 could increase nitrogen-fixing bacteria in root system, which had potential value for biological nitrogen fixation.

**Table 13:** Comparison of major bacterial community composition of soybean-potted root at the family level.

	CK	J	Y	H
*Micromonosporaceae*	31.75 ± 1.45a	10.43 ± 2.64b	5.48 ± 3.59c	5.31 ± 0.94c
*Burkholderiaceaea*	22.53 ± 3.47a	6.16 ± 1.93b	10.53 ± 2.39b	6.82 ± 1.59b
*Rhizobiaceae*	7.10 ± 1.26b	9.20 ± 1.84b	12.77 ± 3.22b	24.81 ± 5.09a
*Sphingomonadaceae*	4.45 ± 2.16c	8.38 ± 2.65bc	10.70 ± 1.12b	15.61 ± 3.21a
*Xanthomonadaceae*	2.79 ± 1.05c	11.07 ± 1.67a	6.90 ± 2.71b	3.92 ± 1.08bc
*Devosiaceae*	3.05 ± 1.74c	6.68 ± 1.31b	5.30 ± 1.48bc	14.11 ± 2.23a
*Streptomycetaceae*	3.70 ± 1.09b	11.67 ± 3.26a	5.04 ± 1.90b	4.65 ± 1.31b

Data are means ± standard errors (*n* = 3). Different letters represent significant difference at *p* < 0.05 (Tukey’s HSD test).

**Table 14:** Comparison of major bacterial community composition of soybean-potted root at the genus level.

	CK	J	Y	H
*Actinoplanes*	25.42 ± 3.74a	4.32 ± 1.80b	3.30 ± 1.63b	1.54 ± 0.53b
*Novosphingobium*	3.90 ± 1.60c	6.99 ± 0.99b	10.31 ± 1.06a	8.75 ± 1.15ab
*Streptomyces*	3.70 ± 1.09b	12.00 ± 2.95a	5.04 ± 1.90b	4.65 ± 1.31b
*Devosia*	2.80 ± 1.53c	6.58 ± 1.28b	4.72 ± 1.17bc	17.19 ± 1.37a
*Allorhizobium-Neorhizobium-Pararhizobium-Rhizobium*	3.61 ± 1.72b	4.50 ± 2.38b	3.41 ± 1.91b	25.41 ± 1.82a

Data are means ± standard errors (*n* = 3). Different letters represent significant difference at *p* < 0.05 (Tukey’s HSD test).

## 4. Discussion

### 4.1. Effects of bacterial inoculants on the growth of potted soybean

Previous studies have reported that the inoculation with PGPR strains can promote the growth of most plants (Singh et al., 2011; Schwachtje et al., 2012; Wang et al., 2021). Inoculation with a single PGPR strain including *P. chlororaphis* H1 and *B. altitudinis* Y1, as well as a mixture of both, promoted soybean growth, increased the aboveground and underground biomass (Table 3), and promoted the root growth (Table 4). Interestingly, we found that nodule number in H group is higher than other groups (Table 4). Root nodule is a recognized nitrogen-fixing structure of leguminous plants. It is a symbiotic structure formed by rhizobia and leguminous plants, which promotes the yield of leguminous plants and reduces the application of external nitrogen fertilizer (Soumare et al., 2020). Furthermore, our study demonstrated the yield-promoting effects of both combined and single PGPR strains. Soybean yield attributes include the number of pods, number of seed per pod, 100-seed weight and so on (Adeyemi et al., 2020). The complex inoculant exhibited better growth-promoting effect on soybean than the single strain inoculant; this indicated that the complex inoculant was more reliable and effective than the single inoculant for soybean production. Thus, our complex inoculant could improve the growth index of soybean and exhibited a positive effect on soybean production.

### 4.2. Effects of bacterial inoculants on physical and chemical properties of soil

Currently, due to the widespread phosphorus deficiency in soils worldwide, the cost of phosphorus fertilizer is increasing despite the low efficiency of plants in using phosphorus from soil and fertilizer sources, and the complex inoculant we developed have some positive implications for soil improvement. In our study, the available phosphorus (AP) concentrations in potted soybean soils were 13.76 mg/kg in group H, which was significantly higher compared with groups C, J and Y (Table 8). Meanwhile, contents of hydrolyzed nitrogen (AN), available potassium (AK) and organic carbon (SOC) were higher than the other three groups after inoculation with the composite bacterial inoculum. In addition, the bulk density, which reflects the degree of soil compaction and maturation, also decreased to 0.98, which was the lowest among the four groups. These results indicate that the application of microbial agents could improve soil physicochemical properties and increase available nutrients. One possible reason for these results is that the PGPR strains used for microbial preparations have the two PGPR strains had the characteristics of phosphorus solubilization, which colonize and grow in the soil and then induce metabolic processes that are effective in directly desorbing and mineralizing precipitated calcium (Ca) phosphate, both inorganic and organic phosphorus (Richardson and Simpson, 2011). Specific reason remains to be further research.

### 4.3. Effects of bacterial inoculants on the bacterial community structure in rhizosphere

The diversity of soil bacterial community can be used to some extent as an indicator of soil ecosystem stability and health (Doran and Zeiss, 2000). As demonstrated in previous studies, the addition of microbial inoculants can significantly change the structure of bacterial communities (Piromyou et al., 2013) in the soil and suppress plant diseases (Qiu et al., 2012; Shen et al., 2015; Chen et al., 2016). To further explore microbial diversity, we analyzed potted soybean rhizosphere soils treated with bacterial inoculants and found that the total OTU number and α-diversity indices (Shannon and Simpson index) of rhizosphere in the H group were significantly higher (*p* < 0.05) than those in groups J and Y, but slightly lower than those in group CK. This result was consistent with the results of Wei et al. (2020), suggesting that the addition of *Pseudomonas* could reduce soil microbial diversity and richness. Interestingly, mixing *Pseudomonas* and *Bacillus* increased microbial diversity in H group.

In our study, we also analyzed the top five species at the level of known bacterial phylun and found community structure of group H was similar to the group CK while in group J was similar to that in group Y (Table 10). The relative abundance of *Firmicutes* and *Actinobacteria* increased in H group compared with CK and the abundance of *Proteobacteria* decreased. It is known that *Firmicutes* phylum contains bacteria that produce spores that are resistant to extreme environments (Galperin, 2013), *Bacillus* belongs to *Firmicutes*, so the relative abundance of *Firmicutes* in Group Y was the highest, which *Geobacillus* and *Bacillus* were the most abundant. By analyzing the genus level, it was found that the reason for the increased *Proteobacteria* was attributed to the increase of *Luteimonas*, *Steroidobacter* and *Pseudomonas*. Besides, we found *Actinobacteria* in H group was obviously improved than other groups. *Actinobacteria* are the source of two-thirds of natural antibiotics, antiparasitics, antifungal agents, and immunosuppressive agents (Demain and Sanchez, 2009). At the genus level, the relative abundance of *Luteimonas* was significantly higher in the H group than in the CK, J, and Y groups. They survive as endophytes in plant tissues and contribute to nutrient assimilation and growth (Jose et al., 2014). In addition, they can survive in extreme environments and drive nutrient cycling under harsh conditions (Bull and Goodfellow, 2019). The metabolites of *Luteimonas* have antibacterial effect and inhibitory effect on *Escherichia coli* and *Staphylococcus aureus*. Interestingly, these two bacteria in group H are higher than those in group CK, J and Y. The soybean growth of group H was the best, possibly because the presence of two antibacterial microorganisms enhanced the resistance to pests and diseases.

### 4.4. Effects of bacterial inoculants on the bacterial community structure in root

Besides, we analyzed microbial population in soybean root in the CK, J, Y, and H group at the level of bacterial family and genus.

Ccompared with CK in control group (Figure 3A), the relative abundance of *Xanthomonadaceae*, *Sphingomonadaceae*, *Devosiaceae*, and *Rhizobiaceae* in group H increased, while *Burkholderaceae* decreased (Table 13). *Paraburkholderia* have similar functions as rhizobia to promote nitrogen fixation and nodulation, and have beneficial growth promoting effects on legumes (Dobritsa et al., 2017). Compared with J and Y, the beneficial bacteria *Xanthomonadaceae* and *Devosiaceae* and *Rhizobiaceae* were significantly increased. *Xanthomonadaceae* can produce antibiotics and extracellular enzymes, degrade the cell walls of fungi and nematodes, and have a strong inhibitory effect on *Hemithromycetes*, *Ascomycetes*, *Basidiomycetes*, and *Oomycetes* (Da Silva et al., 2002). *Sphingomonadaceae*, which is associated with nitrogen and carbon cycling in soil (Luo et al., 2018), increased in soils treated with single inoculant J and Y and mixed group H. Rhizobia inoculants have been used as agricultural products for more than 100 years and for decades it was thought that only rhizobia could promote biological nitrogen fixation. However, a series of α-β-and γ-proteobacteria genera have recently been found to be involved in nitrogen fixation from legume nodules, mainly including *Pantoea*, *Burkholderia*, *Serratia*, *Pseudomonas*, *Bacillus* and *Enterobacter* (Martinez-Hidalgo and Hirsch, 2017; Gopalakrishnan et al., 2018). Some species of *Devosiaceae* have been shown to have similar effects as rhizobia and are a beneficial bacterial population for legumes (Rivas et al., 2003). The family *Pseudomonadaceae* is also famous for its many species that have been proved to be effective in controlling pests and diseases (Ma et al., 2016).

The growth promoting bacteria changed from a single *Burkholderiaceae* family to a variety of growth promoting bacteria interacting with each other to promote plant growth, indicating that the use of our composite inoculum can improve the structure of root bacterial community and increase the bacterial community beneficial to plant growth. In addition, at the genus level, compared with CK, H significantly increased the evenness and richness of species, with no significant difference in the content of several major species, and decreased the content of *Actinoplanes*. The contents of *Allorhizobium-Neorhizobium-Pararhizobium-Rhizobium*, *Devosia* and *Pseudomonas* were significantly increased (Figure 3B). Both at the genus level and the family level showed that the composite inoculum could increase the evenness and richness of root species, and increase the abundance of beneficial bacteria. Therefore, our composite inoculum has a certain potential to regulate microbial population structure and increase beneficial bacterial species in soybean root.

## 5. Conclusion

By identifying two strains, *Pseudomonas chlororaphis* H1 and *Bacillus altitudinis* Y1, which have been shown to have high efficiency in phosphorus solubilization and IAA production, further studies were conducted. After inoculation of soybean plants with two single and combined inoculants prepared from these two strains, the growth indexes and yield of soybean increased significantly. Moreover, physicochemical properties of soil were the highest after inoculation with H inoculant, indicating the value of H inoculant for soil improvement. In addition, the bacterial community structure after the H treatment was similar to that of the control group, whereas the single strain inoculant groups exhibited significant differences in the bacterial community structure compared with that of the native soil and root, and the relative abundance of the indigenous beneficial bacteria, *Luteimonas*, *Allorhizobium-Neorhizobium-Pararhizobium-Rhizobium*, *Devosia* and *Pseudomonas* increased significantly in H treatment. In conclusion, this study reported that both single and complex PGPR strain inoculation could alter the structure of rhizosphere and root endophytic bacterial communities, leading to an increase of the non-native bacterial populations. However, that of a composite bacterial incolum H might better maintain the original bacterial population structure and less damage to the original soil microecology than single inoculants, significantly increasing the original beneficial bacterial populations in rhizosphere and plant and producing a better probiotic effect. In addition, the composite inoculum used in this study showed the potential feasibility of the mixed use of *Bacillus* and *Pseudomonas*. As an inoculant, it had positive significance for promoting soybean growth, increasing soybean yield and improving soil nutrient status, which is valuable for solving the problem of insufficient soybean yield.

## Data availability statement

The original contributions presented in the study are included in the article/supplementary material, further inquiries can be directed to the corresponding author.

## Author contributions

WZ and GM contributed to conceptualization, designed the experiments, performed the experiments, analyzed the data, and wrote the manuscript. HY and JZ contributed to clinical advices, edited the manuscript, and supervised the study. All authors have read and agreed to the published version of the manuscript.

## Conflict of interest

The authors declare that the research was conducted in the absence of any commercial or financial relationships that could be construed as a potential conflict of interest.

## Publisher’s note

All claims expressed in this article are solely those of the authors and do not necessarily represent those of their affiliated organizations, or those of the publisher, the editors and the reviewers. Any product that may be evaluated in this article, or claim that may be made by its manufacturer, is not guaranteed or endorsed by the publisher.
